# Novel non-parametric models to estimate evolutionary rates and divergence times from heterochronous sequence data

**DOI:** 10.1186/s12862-014-0163-6

**Published:** 2014-07-24

**Authors:** Mathieu Fourment, Edward C Holmes

**Affiliations:** 1 Marie Bashir Institute for Infectious Diseases and Biosecurity, Charles Perkins Centre, School of Biological Sciences and Sydney Medical School, The University of Sydney, Sydney, Australia

**Keywords:** ᅟ

## Abstract

**Background:**

Early methods for estimating divergence times from gene sequence data relied on the assumption of a molecular clock. More sophisticated methods were created to model rate variation and used auto-correlation of rates, local clocks, or the so called “uncorrelated relaxed clock” where substitution rates are assumed to be drawn from a parametric distribution. In the case of Bayesian inference methods the impact of the prior on branching times is not clearly understood, and if the amount of data is limited the posterior could be strongly influenced by the prior.

**Results:**

We develop a maximum likelihood method – Physher – that uses local or discrete clocks to estimate evolutionary rates and divergence times from heterochronous sequence data. Using two empirical data sets we show that our discrete clock estimates are similar to those obtained by other methods, and that Physher outperformed some methods in the estimation of the root age of an influenza virus data set. A simulation analysis suggests that Physher can outperform a Bayesian method when the real topology contains two long branches below the root node, even when evolution is strongly clock-like.

**Conclusions:**

These results suggest it is advisable to use a variety of methods to estimate evolutionary rates and divergence times from heterochronous sequence data. Physher and the associated data sets used here are available online at http://code.google.com/p/physher/.

## Background

Accurately estimating evolutionary (substitution) rates and divergence times is central to revealing the timing, patterns, and processes of molecular evolution. Several methods have been developed to co-estimate evolutionary rates and divergence times along a phylogeny or sets of phylogenies. The earliest and easiest method was to assume a strict molecular clock (i.e. homogenous rate among lineages). Although convenient, the assumption of a “strict” molecular clock is often violated, such that more complex models are required that are able to integrate rate variation among lineages. Sanderson [1] addressed this problem using non-parametric rate smoothing and by assuming that rates of consecutive lineages are correlated. Later, he adopted a maximum likelihood framework and used penalized likelihood to investigate different levels of rate correlation, and a penalty multiplier to achieve a trade-off between rate autocorrelation and goodness-of-fit [2]. Thorne, Kishino and Painter [3] further explored the idea of auto-correlation between rates using a Bayesian framework and Markov chain Monte Carlo (MCMC). To accelerate their calculations, the topology is assumed to be known and the likelihood is approximated using a multivariate normal density. The MCMC used is based on Metropolis-Hastings algorithms, which are less efficient than other implementations such as Gibbs sampling [4]. Hence, Lartillot [4] proposed an efficient Gibbs sampler using data augmentation and prior conjugacy, which was extended by Guindon [5] who also used an approximation to the likelihood function. Yang [6] employed maximum likelihood and a heuristic rate smoothing approach to calculate rates and classify lineages into rate classes. This *ad hoc* procedure was further improved by Aris-Brosou [7] using more advanced clustering methods that allow the estimation of the number of rates.

In practice, however, there is no guarantee that evolutionary rates are autocorrelated. In addition, in the case of rapidly evolving organisms such as RNA viruses measurable evolution occurs across the sampling period, such that ‘heterochronous’ sequences sampled at different time-points provide valuable information about evolutionary rates. An alternative approach is therefore to allow rates to vary freely along a phylogeny, and to explicitly incorporate times of sampling. Drummond and colleagues [8] presented an ‘uncorrelated’ relaxed clock model in which rates are drawn independently from an underlying parametric distribution, such as lognormal or exponential. Although rates are not necessarily distributed according to a probability distribution, this approach greatly reduces the number of parameters. Another way of modelling uncorrelated rates is to assume clock-like behaviour within a particular lineage. The main difficulty with this approach is to find the number and the distribution of these local clocks on a phylogeny, although Bayesian stochastic search variable selection has now been used in this context [9]. More recently Heath [10] proposed a model where lineages are assigned a substitution rate value according to the Dirichlet process prior.

Herein, we propose a simple maximum likelihood-based approach to infer substitution rates and divergence times from heterochronous nucleotide sequences. Given a rooted tree, rate variation among lineages is modelled using either local (LC) or discrete (DC) clocks. Our definition of a ‘local clock’ is the same as that used previously [9] and assumes that while the substitution rate may vary across a phylogeny, some adjacent lineages evolve at the same rate. In contrast, the ‘discrete clock’ model assumes that a number of substitution rate categories are assigned to lineages without assuming autocorrelation and where lineages that are not adjacent are able to share a rate category. We devised a heuristic approach using a greedy algorithm to infer the distribution of local clocks along a phylogeny, referred to here as the Heuristic Local Clock (HLC) algorithm. The estimates of the best model can be fed to a genetic algorithm (GA) to re-estimate the rates and local clock positions, and calculate model-averaged estimates of the substitution rate and time parameters (i.e. GALC). Similarly, we present a GA to determine the number and allocation of rate categories under a discrete clock model (GADC). The greedy algorithm and GAs are optimized to run efficiently on a distributed computing environment using OpenMP. Finally, we demonstrate the efficiency of the program using data sets of human influenza viruses and simulated data sets.

## Methods

### Models of rate variation among lineages

Given *n* nucleotide sequences and a rooted phylogeny with *N = 2n-2* branches, we set out to model rate heterogeneity along lineages using local clocks or a discrete distribution of rates.

We define a local clock on a phylogeny as a monophyletic group where every lineage evolves at exactly the same substitution rate. This definition assumes the existence of another clock (e.g. ‘global’ clock) for lineages that are not assigned a local clock. We model each local clock and the global clock as independent rate classes. In the absence of local clocks, the model corresponds to a strict clock; the other extreme is to have one rate per branch, leading to an over-parameterized model. The optimization challenge for this problem is two-fold: finding the number and location of the local clocks along a phylogeny (discrete optimization) and estimating rates and ages of internal nodes (continuous optimization). The difficulty in using this type of clock lies in the discrete optimization component because of the size of the combinatorial search space. With *2*^*2n-2*^ rate combinations, it is impossible to adopt a brute force approach and heuristic algorithms must be used instead.

We also consider an alternative and more flexible parameterization to local clocks by assigning independent substitution rate classes to lineages to model lineage-specific rate heterogeneity. Unlike the local clock model, this discrete clock model allows non-adjacent lineages to evolve under the same substitution rate. Local clocks are therefore a special case of discrete clocks. Given a fixed number of rate categories *k*, the parameters of the discrete substitution model is a vector of rates ***r*** = (*r*_1_, *r*_2_, …, *r*_*k*_) and a vector of rate-class assignments for each lineage ***c*** = (*c*_1_, *c*_2_, …, *c*_2*n* − 2_) where *c*_*i*_ ∈ 1 … *k*. Using an appropriate ordering of the *2n-2* branches, each element of vector ***c*** represents the class assignment of a branch. In our implementation, the number of rate categories is not fixed and is co-estimated with node heights and vector ***r*** from the data. The number of combinations for the discrete clock algorithm is also computationally difficult and warrants the use of approximation methods. The number of combinations is described by the Bell number $B_{N}=\sum_{k=0}^{N}S\left(N,k\right)$ where *N* is the number of branches and

(1) $$S\left(N,k\right)=\frac{1}{k!}\sum_{i=0}^{k-1}{\left(-1\right)}^{i}\left(\begin{array}{c} k\\ i \end{array}\right){\left(k-i\right)}^{N}$$

is the Stirling number of the second kind. For example, for a relatively small data set of 20 sequences, there are approximately 7 × 10^32^ possible combinations.

### Algorithms

One solution to search the high-dimensional parameter space of the local and discrete clock models without using an MCMC is to use a genetic algorithm (GA). GAs belong to a class of evolutionary algorithms that simulate natural selection in a population of individuals to solve an optimization problem. Most algorithms require encoding solutions in a vector and are therefore well suited to discrete optimization. Each individual represents a candidate solution from which a fitness score can be calculated. Individuals with high fitness, and therefore a promising solution, are more likely to be selected during a stochastic step (mating or recombination) to form the next generation. In analogy to biological processes, candidate solutions randomly mutate to promote diversity within the population. GAs are therefore generational processes that stochastically move populations around a fitness landscape. The simplest form of genetic algorithm requires a fitness function and three operators: recombination, selection and mutation.

We implemented the GA as a generational genetic search algorithm CHC GA [11]; this approach was previously applied to the detection of both recombination [12] and natural selection [13]. The population size of CHC GAs are usually smaller than traditional GAs and use an elitist selection operator that always allows the fittest individual to be selected for the next generation. In our analyses, we used a fixed population size of 30 individuals. In addition, it uses a highly disruptive recombination operator that generates a new individual with a solution that contains half the solution from the first parent and the other half from the second parent. The mutation step is only triggered when the diversity of the population is below a fixed threshold by mutating a fixed number of elements of the solution vector. Given a fixed number of rate classes, the fitness function is simply the likelihood score since every model has the same number of parameters. The mutation and recombination operators for the local and discrete clocks are different and will be presented in the next sections.

Finding the number of rate classes is more challenging and is not easily addressed with GAs, as it requires jumping between parameter spaces with different dimensions. We use an iterative approach, starting with two rate classes and increment the number of classes until the addition of a rate class stops improving the fitness of the model. During each iteration, a new population is evolved, leading to the fittest model that will be compared to the next iteration. To test whether the addition of a rate class improves the fitness of the model, we assess the goodness of fit of each model using the Akaike Information Criterion with a correction for small sample sizes (*AICc*; the lowest *AICc* represents the best model):

(2) $$\mathrm{AICc}=-2Ln\hat{L}+2k+\frac{2k\left(k+1\right)}{s-k-1}$$

where $Ln\hat{L}$ is the maximum likelihood estimate under the model of interest, *k* is the number of parameters and *s* is the sample size. We set the sample size as the number of site patterns in the alignment.

In the local clock model, each individual of the GA population is represented as a vector where each element maps a local clock to a branch in the phylogenetic tree. If a branch is assigned a local clock, its descendants will belong to the same local clock unless they are assigned a new local clock. The mutation operator randomly changes the location of the local clock. The recombination operator selects two individuals from the population and generates a new individual by randomly choosing the value of one of the parents for each element of the vector state. In the current implementation of the GA, both operators do not allow a local clock to be assigned more than once. The most extreme case is when there is only one local clock: the vector is of size one and recombination has no effect. For models containing a few local clocks, the diversity of the population drops under the threshold after the first round of mating. To escape this problem a mutation rate close to 100% is needed, defeating the purpose of evolutionary algorithms. A solution to this problem would be to start the GA with several local clocks, but determining an appropriate number of local clocks is difficult. Alternatively, we could employ exhaustive search on all the combinations of local clocks but this is only possible for small data sets. We implemented a greedy algorithm that starts with one local clock, fits in turn a local clock to each branch, and retains the location of the local clock that yields the best likelihood. In each subsequent iteration, another local clock is considered on each branch that does not have a local clock already assigned. The algorithm stops either when the addition of a parameter does not improve the fitness of the model based the *AICc* or when the number of clocks exceeds a user-defined threshold *T* (typically *T <<N-1*). Therefore, the algorithm will evaluate a maximum $\sum_{i=1}^{T}T-i$ models.

The best model found by the greedy search will have enough parameters for a GA to more efficiently sample the parameter space without the convergence issue discussed earlier. Hence, we use the fittest model of the greedy search to seed the GA. This approach allows us to assess other combinations that were not previously tested. Since models with different numbers of classes are not necessarily nested we also compute *AICc* to compare models.

Given a fixed number of rate classes, the encoding of each individual of the GA in the discrete clock model is represented as vector of size *N* where each element represents the class assignment of a branch. The number of rate classes can take any value between 1 (strict clock) and *N* (one rate per branch), although the number of classes should be much smaller than *N* in order to limit model complexity as mentioned earlier. The recombination operator for the discrete model simply selects two individuals from the population and generates a new individual by randomly choosing the value of one of the parents for each element of the vector state. During the mutation step, each element of the vector is subject to switching to a different class assignment with fixed probability. As in the GA for local clocks we adopt a forward selection approach, starting with two rate classes and increment by one the number of rate classes until the *AICc* stops decreasing.

Finding the combination of rate classes using the greedy algorithm described above is substantially more difficult as the combinatorial complexity increases; therefore, we employ a CHC-GA to search through the parameter space.

### Model averaging and confidence intervals

Uncertainty is inherent to selecting the best model. As a consequence it might be desirable to model average estimated parameters across all the models *M* explored by the GA. First, we need to compute for each model *M*_*i*_ the *AIC* difference (Δ_i_) between the best model and itself such as Δ_*i*_ = *AIC*_*min*_ − *AIC*_*i*_, where *AIC*_*min*_ is the *AIC* of the best model. Akaike weights are now calculated as:

(3) $$w_{i}=\frac{e^{\left(\Delta_{i}/2\right)}}{\sum_{r=1}^{M}e^{\left(\Delta_{r}/2\right)}}$$

where the numerator is the relative likelihood of model *M*_*i*_ and the denominator is a normalization term. The relative likelihood can be interpreted as the relative probability that the *i*^*th*^ model minimizes the information loss. Once the models are sorted in decreasing order, we can obtain a confidence set of models by summing the weights from the largest to the lowest until the sum is (1-α) where α is the significance level. Descriptive statistics such as mean and quantiles can be applied to each parameter using the models belonging to this set.

To investigate the uncertainty of the point estimates under a strict clock and a local clock model with the greedy algorithm, we calculated confidence intervals (CI) using the non-parametric percentile bootstrap method [14]. The percentile bootstrap uses the empirical quantiles from the bootstrap distribution of the parameter *θ* to calculate the confidence interval $\left(\theta_{\left(\alpha /2\right)}^{*};\theta_{\left(1-\alpha /2\right)}^{*}\right)$ where *α* is the significance level and $\theta_{\left(\alpha /2\right)}^{*}$ and $\theta_{\left(1-\alpha /2\right)}^{*}$ are the *α*/2 and 1 − *α*/2 percentile of the bootstrap coefficients θ*.

### Program workflow

The first step that is common to the GA and greedy algorithms is to estimate the branch lengths (in term of expected number of substitution per site) of the phylogeny and the parameters of the substitution model by maximum likelihood. The likelihood of this parameter rich non-clock model will give us an upper-bound for the likelihood of the global, local and discrete clock models. The parameters of the substitution rate matrix are fixed during the rest of the algorithm as we make the assumption that nucleotide frequencies and relative rates are not affected by explicitly modelling substitution rates. Next, we estimate the age of *n-1* internal nodes and the substitution rate under a strict clock model. This simplistic model, if violated, gives us the lower-bound in term of likelihood and the starting point for statistically testing the introduction of rate heterogeneity in the model. Because we are explicitly dealing with heterochronous sequences, every taxon must be assigned a fixed date.

### Implementation

Since the GA and greedy search algorithms presented in this paper are embarrassingly parallel problems, we implemented the program within the OpenMP framework to run on distributed systems. The evaluation of the likelihood for each configuration of clocks was therefore computed by a single thread. Finally, the likelihood is calculated using Felsenstein’s pruning algorithm [15] and is optimised with SSE-based SIMD vectorization. These methods are implemented in a C program called Physher. Simulated data sets were generated with a custom program that uses a library shared with Physher.

### Data sets and analysis

**Figure 1:**
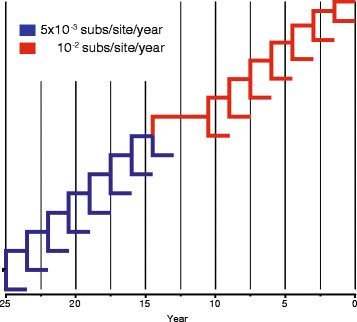
**Phylogeny used for simulating data sets under a local clock model.** Lineages are coloured according to their substitution rates.

We applied our algorithms to two data sets of heterochronous viral nucleotide sequences. The first data set comprises an alignment of 69 human influenza A/H3N2 virus haemagglutinin (HA) sequences (987 nt in length) isolated between 1981 and 1998. The evolutionary rates and time to the most recent ancestors (tMRCAs) of this data set was previously investigated using a random local clock method [9] with a Bayesian MCMC approach implemented in BEAST [16]. As in the original study, the alignment was analysed using strict, local and discrete clocks implemented in Physher under the HKY + Γ_4_ substitution model that incorporates gamma-distributed rate variation among sites (4 rate classes). We reanalysed the data using BEAST with either an uncorrelated lognormal relaxed-clock (UCLN) or a random local clock model (RLC) and calculated the Bayes factor (BF) using the path sampling method [17] to compare competing models. We performed a series of simulations to assess the effect of stochastic noise over the phylogenetic signal. Accordingly, data sets of 1000, 2000 and 10,000 nucleotides (nt) in length were simulated along a ladder-like tree containing 16 taxa that represents a simplified version of the influenza A data set (Figure 1). The root age is 25 years, the global rate is set to 5 × 10^−3^ subs/site/year and a local clock is set in the middle of the tree inducing a rate shift with rate equal to 10^−2^ subs/site/year. 50 replicates were simulated under the HKY model with equal nucleotide frequencies, a transition to transversion rate ratio *κ* = 3, and a gamma shape *α* = 0.5. These data sets were analysed using the RLC and UCLN with a constant size coalescent model and an exponential prior on the rate with mean 8 × 10^−3^ subs/site/year using BEAST. The GADC and HLC models were used in Physher.

The second data set comprising 265 full-length HA genes (1382 nt in length) of influenza B virus, containing sequences from the co-circulating Yamagata and Victoria lineages isolated between 2002 and 2013. Each taxon was calibrated using year, month, and day of isolation. The substitution rate and tMRCA were estimated under a GTR + Γ_4_ substitution model using strict and discrete clocks with Physher. The data was also analysed using BEAST [16] under a strict clock and an uncorrelated lognormal relaxed clock and with both constant population size and Bayesian skyride tree priors. As previous analyses [18] estimated the substitution rate for influenza B virus to be 1 – 3 × 10^−3^ nucleotide substitutions per site per year (subs/site/year), we used an exponential prior with mean 3 × 10^−3^ subs/site/year on the mean of the lognormal distribution and strict rate parameter. We did not use any prior assumption on the age of the root.

Influenza B virus data sets were also simulated under a strict molecular clock using divergence times, substitution rates and other substitution parameters estimated by Physher. These data sets are effectively parametric bootstraps with a fixed topology. The parameters of importance in this simulation are the substitution rate, for which the Physher estimate was 1.4 × 10^−3^ subs/site/year and the age of the root (*t* = 1967) (see Results). In this case we know that the root predates, or should be close, to 1972 since the two main lineages became antigenically distinct in the 1970’s [19] and the earliest virus that emerged after the split was sampled in 1972. We analysed 50 replicates with Physher and BEAST using a strict clock. The BEAST analyses were performed using a constant population size, and both the Bayesian skyride [20] and the constant rate birth-death methods [21]. To investigate the effect of allowing rate variation among lineages we also analysed the replicates using an uncorrelated relaxed lognormal prior. In this case we used an exponential prior with mean 2 × 10^−3^ subs/site/year on the mean of the lognormal distribution and strict rate parameter. We performed at least two analyses of 20 million generations each and used Tracer [22] to assess the stationarity of the chain and to discard an appropriate number of generations. Support for the substitution rate and root age was given by 95% highest posterior density intervals. Confidence intervals were derived using the percentile bootstrap method with 200 replicates. We also inferred the rate and root age, using a fixed topology, by regressing the expected number of substitution per site from the root to each tip from the maximum likelihood tree against sampling times with Physher. To obtain a rooted a tree, a node (the root) needs to be inserted in the unrooted tree by splitting a branch in two. We estimate the location of the root as the location that maximizes the coefficient of correlation between time and the expected number of substitutions. The rate and root age are defined as the slope and intercept of the regression line, respectively.

**Figure 2:**
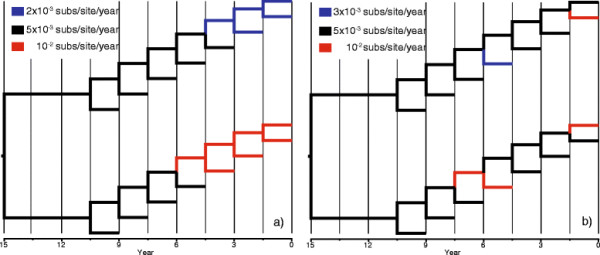
**Phylogenies used for simulating data sets under different clock models.** Lineages are coloured according to their substitution rates. **(a)** Phylogeny with two local clocks. **(b)** Phylogeny with three rate classes.

To validate our algorithms we simulated two series of data sets of 2000 nt in length using a local clock model with two local clocks and a discrete clock with three rate classes (Figure 2). The age of the root was 15 years and the rates were 3 × 10^−3^, 5 × 10^−3^, and 10^−2^ subs/site/year. We analysed 50 replicates of each scheme using Physher with a strict, local, and discrete clock and the confidence intervals of the root were derived for the root age using the methods described previously.

## Results

### Influenza A virus

#### Empirical data

We used our local and discrete clock methods to investigate rate heterogeneity of the influenza A virus data set (Additional file 1: Figure S1) by comparing the *AICc* of a strict clock to models that allow rate heterogeneity among lineages. The greedy algorithm found that ten local clocks were the best-fit to the data, improving the log-likelihood from −4159.51 (*AICc* = 8484.73) under the strict molecular clock to −4118.58 (*AICc* = 8420.17) under the local clock. Subsequently, we ran a GA starting with ten local clocks to check that the greedy search did not get trapped in local optima, but this approach failed to find a better model.

We also modelled rate variation with a discrete clock and allocated the rate categories to each lineage using a genetic algorithm. We started the algorithm with two rate classes and incremented the number of classes until the fit of the model stopped improving based on the *AICc*. The algorithm suggested that the maximum likelihood is −4104.59 and that the optimal number of rate classes is four. The *AICc* value of the GADC model (*AICc* = 8376.4) was smaller than the HLC model. The relative likelihood of the HLC model is about 10^−23^, suggesting that the GADC model is about 10^23^ times more likely to be correct than the HLC model.

**Figure 3:**
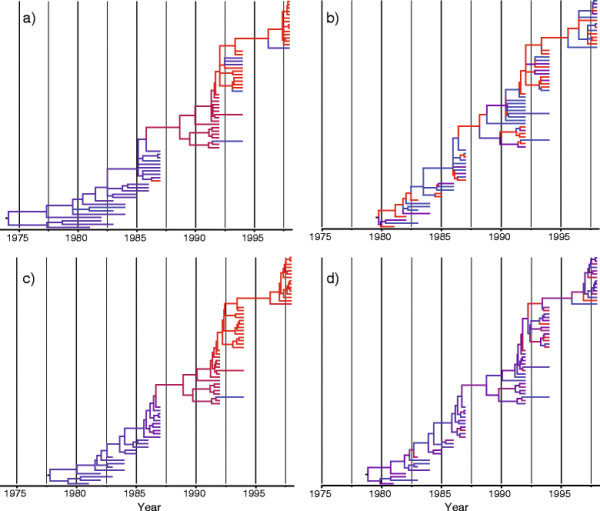
**Influenza phylogenies estimated using Bayesian and maximum likelihood methods.** Branches are coloured according to their rates using a gradient from blue (low rate) to red (high rate). **(a)** Maximum likelihood inference using a local clock model. **(b)** Maximum likelihood inference using a discrete clock model. **(c)** Bayesian inference using a local clock model. **(d)** Bayesian inference using an uncorrelated lognormal relaxed clock.

The mode of the posterior distribution of the number of local clocks reported by Drummond and Suchard [9] was significantly lower than our estimate suggesting that two local clocks were appropriate for this data set. The important difference between our estimates is probably due to the specification of the prior that placed 50% of the mass on a unique clock. Although the root height estimates inferred using our two methods (HLC: 1974.6, CI: 1953.2-1979.27; GADC: 1979.7, CI: 1978.5-1980) are in agreement with their estimate [9] (RLC: 1979, BCI: 1977.98-1979.93, UCLN: 1977.8, BCI: 1975.8-1979.6), the UCLN and GADC models show no obvious rate shift (Figure 3), and rates between each branch and its parent are uncorrelated (r = −5 × 10^−3^; p-value = 0.95). To check whether this shift was caused by the specification of the local clock model we reanalysed the same data set with an uncorrelated lognormal relaxed-clock model using BEAST. If the viral genomes underwent a phase of accelerated mutation rate the UCLN model would be able to recover the same pattern. The distribution over the rates using UCLN does not show this trend although the root height estimate is similar (1978.83, BCI: 1977.06-1980.25). The Bayes factor calculated using the path sampling method (log BF = −8.4) suggests the UCLN model (log marginal likelihood = −4384.83) is strongly preferred to the local clock model (log marginal likelihood = −4388.25).

#### Simulations

**Figure 4:**
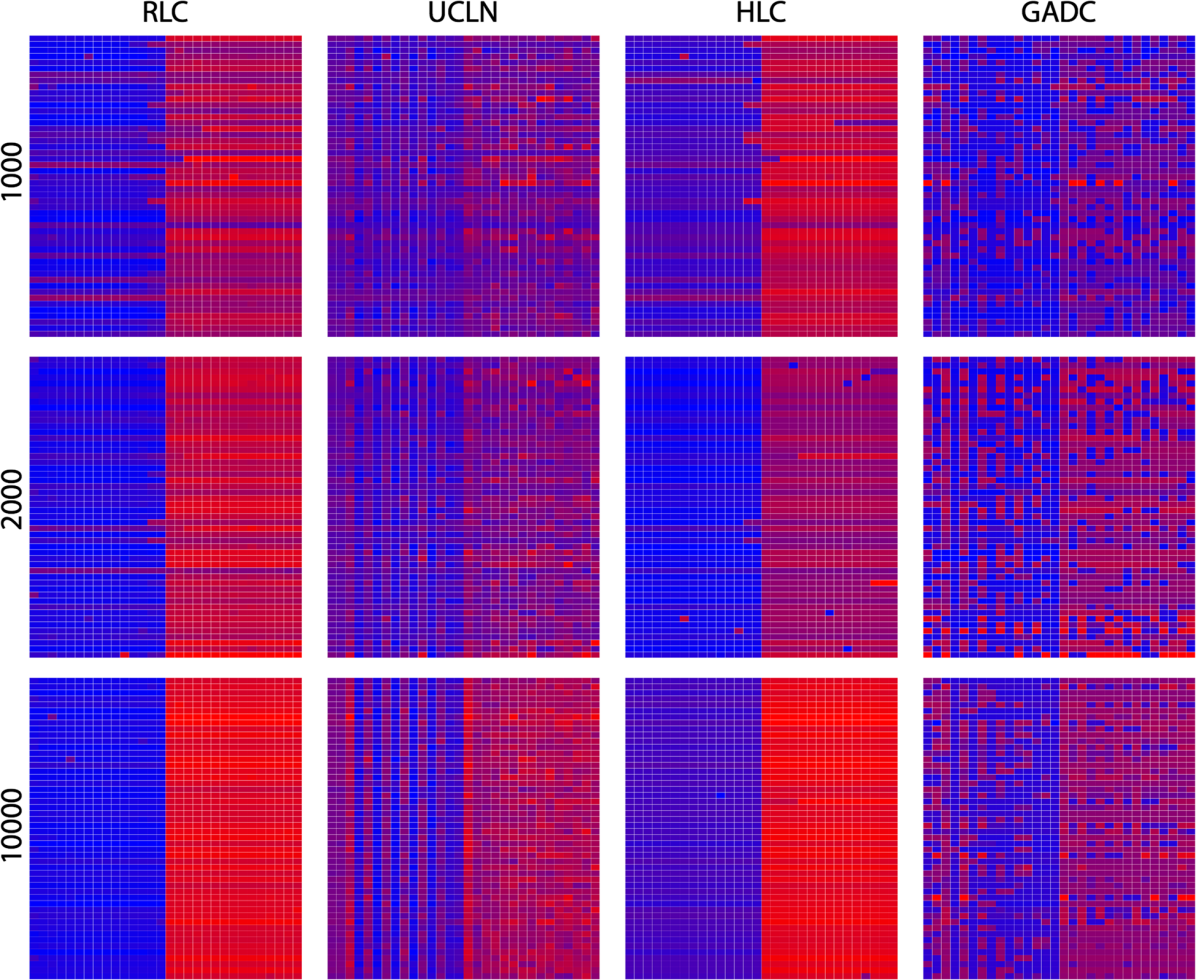
**Substitution rates for simulated data sets with a rate shift.** Substitution rate variation in simulated data sets of length 1000, 2000, and 10000 nt using random local (RLC) and relaxed lognormal (UCLN) clock models implemented in BEAST and local (HLC) and discrete (GADC) clock models implemented in Physher. Each row represents a replicate and each column represent a branch where the rightmost cell is a branch leading to latest taxon and the leftmost cell is a branch near the root. Each cell maps a branch is coloured according to its substitution rate using a gradient from blue (low rate) to red (high rate).

We simulated 50 data sets of different length with a rate shift occurring in the middle branch of a completely asymmetric phylogeny (Figure 1). Each panel of Figure 4 depicts rate variation using a heatmap for 50 replicates where each row represents a replicate and each column indexes a branch. Because of the ladder-like nature of the phylogenies, each tree was linearized using a postorder traversal of the tree, allowing visualisation of rate through time. Starting from the left, the first cell is represents the rate (mean for BEAST and maximum likelihood estimate for Physher) for the branch leading to the earliest taxon and the last two cells represent the rates of the branches leading to the youngest. Between these cells, rates at branches leading to internal and external nodes alternate. These plots clearly show a rate shift when a local clock is used with BEAST and Physher. Plots generated with UCLN and discrete clock models show a more nuanced picture where low and high rates tend to blend together.

### Influenza B virus

#### Empirical data

We analysed the influenza B virus data set using Physher under both non-clock (Additional file 2: Figure S2) (log likelihood LnL = −9503.2) and strict clock (LnL = −9585.9) models. To test whether every lineage evolved at the same rate, we used the likelihood ratio test [15]. The comparison of twice the log-likelihood difference with a χ^2^ distribution with 262 degrees of freedom suggests the strict clock model could not be rejected (p-value = 0.99). The estimate of the substitution rate was 1.42 × 10^−3^ subs/site/year with 95% bootstrap confidence interval [1.2 × 10^−3^, 1.49 × 10^−3^]. The divergence time of the two lineages is estimated to be 1966 (95% CI: 1959, 1971). The same data set was reanalysed using BEAST and the mean substitution rate estimate was 2.1 × 10^−3^ subs/site/year (95% BCI: 1.85 × 10^−3^, 2.36 × 10^−3^) under a constant population size coalescent model and 2.72 × 10^−3^ subs/site/year (95% Bayesian CI: 2.4 × 10^−3^, 3 × 10^−3^) using the Bayesian skyride tree prior. Similarly the mean root age estimate was 1978 (95% Bayesian CI: 1973,1984) under a constant population size coalescent model and 1998 (95% Bayesian CI: 1997,1999) using the Bayesian skyride method and hence considerably more recent that the ‘true’ estimate.

#### Simulations

**Figure 5:**
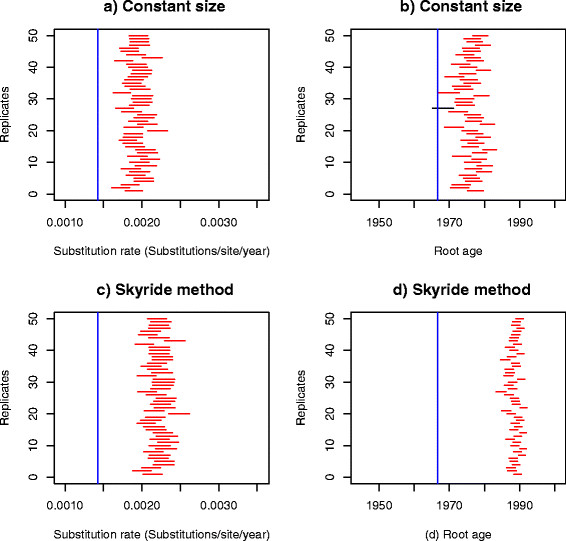
**95% Bayesian confidence intervals of substitution rate and root age using the coalescent prior and a strict molecular clock on 50 simulated data sets.** Parameters are estimated using BEAST for 50 replicates under a strict molecular clock and a coalescent tree prior. Intervals that do not include the true value (blue line) are shown in red. Nucleotide substitution rate **(a)** and root age **(b)** inferred using a constant size tree prior. Nucleotide substitution rate **(c)** and root age **(d)** inferred using the Bayesian skyride method.

**Figure 6:**
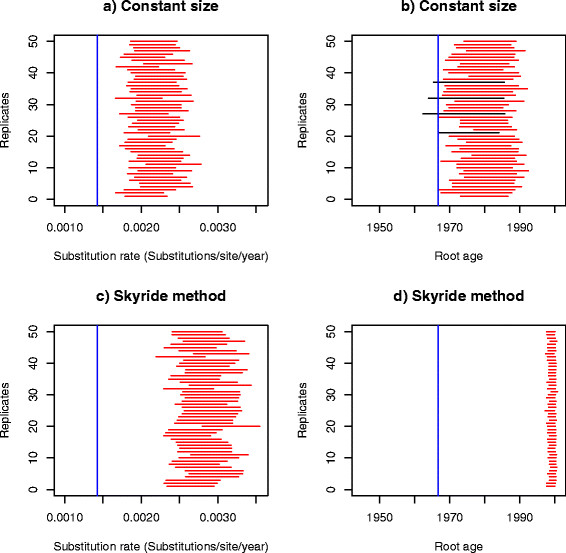
**95% Bayesian confidence intervals of substitution rate and root age using a coalescent prior a nd a relaxed molecular clock on 50 simulated data sets.** Intervals are estimated using BEAST for 50 replicates under a lognormal molecular clock and a coalescent tree prior. Intervals that do not include the true value (blue line) are shown in red. Nucleotide substitution rate **(a)** and root age **(b)** inferred using a constant size tree prior. Nucleotide substitution rate **(c)** and root age **(d)** inferred using the Bayesian skyride method.

**Figure 7:**
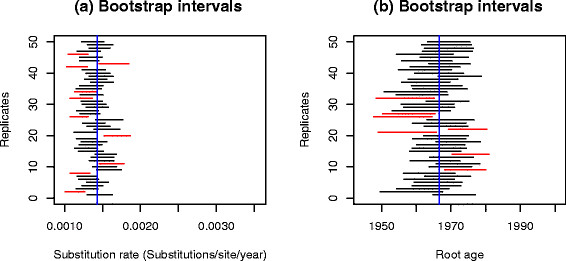
**95% bootstrap confidence intervals of substitution rate and root age using maximum likelihood and a strict molecular clock on 50 simulated data sets.** The nucleotide substitution rate **(a)** and root age **(b)** are inferred using Physher for 50 replicates using a strict molecular clock. Red intervals that do not include the true value (blue line) are shown in red.

We simulated 50 data set replicates using the parameters estimated with Physher in the influenza B analysis and then re-estimated the parameters using Physher and BEAST. We initially used a strict molecular clock with BEAST. Using a constant size coalescent model (Figure 5) none of the Bayesian credible intervals of the substitution rate contained the true rate, and only one interval contained the true root age. The substitution rate was consistently overestimated while the age of the root was consistently underestimated. More striking was that the Bayesian skyride coalescent model underperformed compared to the constant size model, under-estimating the age of the root by about 20 years, as was seen with the empirical data (Figure 6). The same pattern of over-estimation of the rate and underestimation of the root age was observed using a lognormal relaxed clock. Interestingly, the confidence interval of the standard deviation of the lognormal distribution and coefficient of variation did not include 0 using the Bayesian skyride method, suggesting that there was rate variation in the data. Finally, using a birth-death process prior, the rate and root age could not be recovered in any of the replicates (Additional file 3: Figure S3). The bootstrap analysis with Physher showed that 40 intervals contained the true substitution rate and 43 intervals contained the true root age (Figure 7). Finally, the rate and root age estimates using a simple linear regression in Physher are consistent with our maximum likelihood estimates (Additional file 4: Figure S4).

### Additional simulations

Finally, we performed two sets of simulations with two local clocks and three discrete classes. The phylogeny and the rate allocation to lineages are shown in Figure 2. Table 1 shows the number of replicates (out of 50 replicates) for which the true root age lies within the 95% confidence interval. As expected, the strict clock model does not perform well in the presence of rate variation. The local clock model recovered the true root age in every replicate simulated with a local clock and it recovered the true root age in 49 of 50 replicates simulated under a discrete clock model. The discrete clock model recovered the true root age in 48 and 46 replicates in the local and discrete clock data sets. The distribution of the estimated number of rates suggests that the discrete model tends to under-fit the data by assigning two rate classes to most replicates.

**Table 1:** Results of the analysis of simulated data sets

**Clock**	**Two local clocks**		**Three discrete rate classes**	
	**Root age**	**Number of rates**	**Root age**	**Number of rates**
Strict	29	1	33	1
Local	50	2 (4), 3 (34), 4 (10), 5 (2)	49	2 (16), 3 (11), 4 (18), 5 (5)
Discrete	49	2 (35), 3 (15)	46	2 (49), 3 (1)

The table shows the number of replicates (out of 50) for which the true root age lies within the 95% confidence interval estimated by Physher for data sets simulated under two different clock models. The ‘number of rates’ column shows the distribution of the estimated number of rates with their proportion between brackets.

## Discussion

We describe local and discrete clock models to estimate nucleotide substitution rates from heterochronous sequence data sets within a maximum likelihood framework and in the presence of rate heterogeneity among lineages.

The analysis of influenza A virus shows that the discrete clock model fits the data better than the local clock model, as suggested by the Bayes factor calculated using BEAST and the relative likelihood calculated using Physher. Although root age estimates under local and discrete clocks are similar, the relaxed (UCLN) and the discrete clock failed to recover the rate shift pattern that Drummond and Suchard [9] first identified using a random local clock. We performed a series of simulations by generating alignments of 1000, 2000, and 10,000 nt in length with a tree similar to the influenza A virus data set and with a rate shift in the centre of the tree (Figure 1). While the local clock algorithms recovered the location of the rate shift for the shortest alignments, the relaxed and discrete clock model failed to identify the rate shift for data sets of 1000 and 2000 nt. The rate shift only becomes apparent for the data set of 10,000 nt under the discrete model. These simulations therefore confirm that more flexible models cannot recover a single rate shift from 5 × 10^−3^ to 10^−2^ subs/site/year for relatively short alignments. A likely explanation for this discrepancy is that there is not enough data for the UCLN and DC models to recover the true distribution of rate and a certain degree of autocorrelation is needed to correctly identify rate variation.

With limited data, Bayesian-based inference should perform better than the maximum likelihood method if one can use an appropriate prior on the branching times. Unfortunately, it can produce posterior distributions that are heavily influenced by priors, as is probably the case in our simulation study where no coalescent model might be appropriate. The two main classes of prior that are currently used are coalescent processes and the birth-death process. The coalescent process is a function of the effective population size (*N*_*e*_) scaled by the generation time (*τ*), and since no assumptions are made about the generation time in our analyses we refer to this composite parameter (*θ* = *N*_*e*_*τ*) as the relative genetic diversity. The relative size function can be constant, distributed according to a parametric distribution (e.g. exponential distribution) or any function of time. Importantly, in data sets that contain several lineages that evolved concurrently with incomplete sampling of extant taxa (e.g. influenza B virus data set) these priors can produce spurious results. Using simulations it was shown that relaxed clock models can greatly underestimate the age of the root when lineages exhibit strong rate heterogeneity [23]. In the influenza B virus example, the two long branches below the root node (i.e. the ancestors of the Victoria and Yamagata lineages) suggest an initially large relative genetic diversity. With a fluctuating relative diversity the constant coalescent appears to be a poor choice of prior and the Bayesian skyride method should be more appropriate. Indeed, it is striking that the age of the root estimated with BEAST using different priors is significantly younger than the Physher estimate. Hence, we suggest that using a relaxed molecular clock with the Bayesian skyride method should be avoided when a tree contains long branches at the root. In our analyses, the prior appears to overpower the likelihood and is in a favour of a younger root age with a faster rate.

Genetic algorithms appear to be well suited for optimizing the allocation of discrete clocks but their efficiency in the local clock problem is arguable, probably due to the different solution encodings. In the discrete case the individual size is the number of branches (N = *2n-2)*, whereas in the local clock setting the length of each individual is equal to the number of local clocks being investigated. In the influenza A virus data set, we started the GA with ten local clocks. Discrete optimization is notoriously more difficult than its continuous counterpart because functions are generally not convex and because a modification of only one of the variables can significantly change the likelihood of the model.

With respect to performance, the greedy algorithm finished in about 20 minutes on a Mac Pro 3.33 GHz 6-core and the discrete clock GA converged in about two hours for the analysis of the influenza virus A data set. For 20 million cycles, the random local clock ran for approximately 4 hours and the relaxed clock ran for about 5 hours. It is important to highlight that although Physher appears faster than BEAST, Physher optimizes a single topology while BEAST integrate over topologies.

The algorithms described in this paper rely on some common assumptions but differ from other methods in several ways. Several published algorithms [1],[3],[5],[6] use a normal approximation to the costly likelihood function, while Sanderson [2] used a Poisson approximation to describe the mutation process. Some work has also been done on improving the approximate likelihood calculation using parameters transform [24]. To speed-up our inferences we rely instead on parallelization of our algorithms, hence avoiding the assumption that the parameters (product of rate and time) are sufficiently close to the maximum likelihood estimates of the branch lengths inferred in the rate-free analysis. Like many methods we assumed that the rooted tree topology is known; while this is computationally attractive, this assumption is not always realistic. Notably, Aris-Brosou [7] investigated the impact of ignoring topology uncertainty on an empirical data set and revealed no significant differences in age estimates when topology uncertainty was integrated.

The nearly constant substitution rate of the human influenza B virus phylogeny should provide a set of favourable conditions for the estimation of these parameters. Importantly, our simulations suggest that estimating divergence times is difficult if the prior overrides the data even when the substitution rate is constant. Wertheim et al. [25] previously pointed out how rate variation resulted in major discrepancies in the estimation of the age of HIV-1 M group subtypes when each subtype was analysed separately or combined in a total data set of all subtypes. In a similar way to the influenza B virus simulations undertaken here, prior information on the topology could also be a reason for this discrepancy between full phylogeny and sub-tree only estimates. Finally, if a data set contains weak or no temporal signal (i.e. strong rate variation), the estimation of the evolutionary history will be impossible [26].

## Conclusions

We have presented algorithms to estimate evolutionary rates and divergence times from heterochronous gene sequence data without making assumptions about the distribution of rates across a phylogeny. In our analyses the local clock underperformed compared to the discrete clock model, and empirical data that show use of local clock models is only warranted when there is a clear rate shift among lineages. We also show that current Bayesian methods can sometimes fail to recover true node ages and rates due to the specification of the prior.

## Competing interests

The authors declare that they have no competing interests.

## Authors’ contributions

MF conceived and designed the experiments. MF implemented the software Physher. Both authors were involved in the writing of the manuscript. Both authors read and approved the final manuscript.

## Additional files

## Supplementary Material

Additional file 1: **Maximum likelihood phylogeny of the influenza A virus data.** Maximum likelihood tree without the assumption of a molecular clock. Branch lengths depict the expected number of substitution per site. Click here for file

Additional file 2: **Maximum likelihood phylogeny of the influenza B virus data. Maximum likelihood tree without the assumption of a molecular clock.** Branch lengths depict the expected number of substitution per site. Click here for file

Additional file 3: **95% Bayesian confidence intervals of substitution rate and root age using the birth-death model and a strict clock in 50 simulated data sets.** Confidence intervals of the nucleotide substitution rate (a) and root age (a) were inferred using BEAST for 50 replicates using a birth-death model prior on the phylogeny. Intervals that do not include the true value (blue line) are shown in red. Click here for file

Additional file 4: **Linear regression estimates of substitution rate and root age in 50 simulated data sets.** Point estimates of the nucleotide substitution rate (a) and root age (b) using a root to tip linear regression of the expected number of substitutions per site and sampling dates. Click here for file
