# Supplementary figures and images for: Novel non-parametric models to estimate evolutionary rates and divergence times from heterochronous sequence data
Source: BMC Evol Biol. 2014 Jul 24;14:163. doi: 10.1186/s12862-014-0163-6 (PMC4222489; doi:10.1186/s12862-014-0163-6)

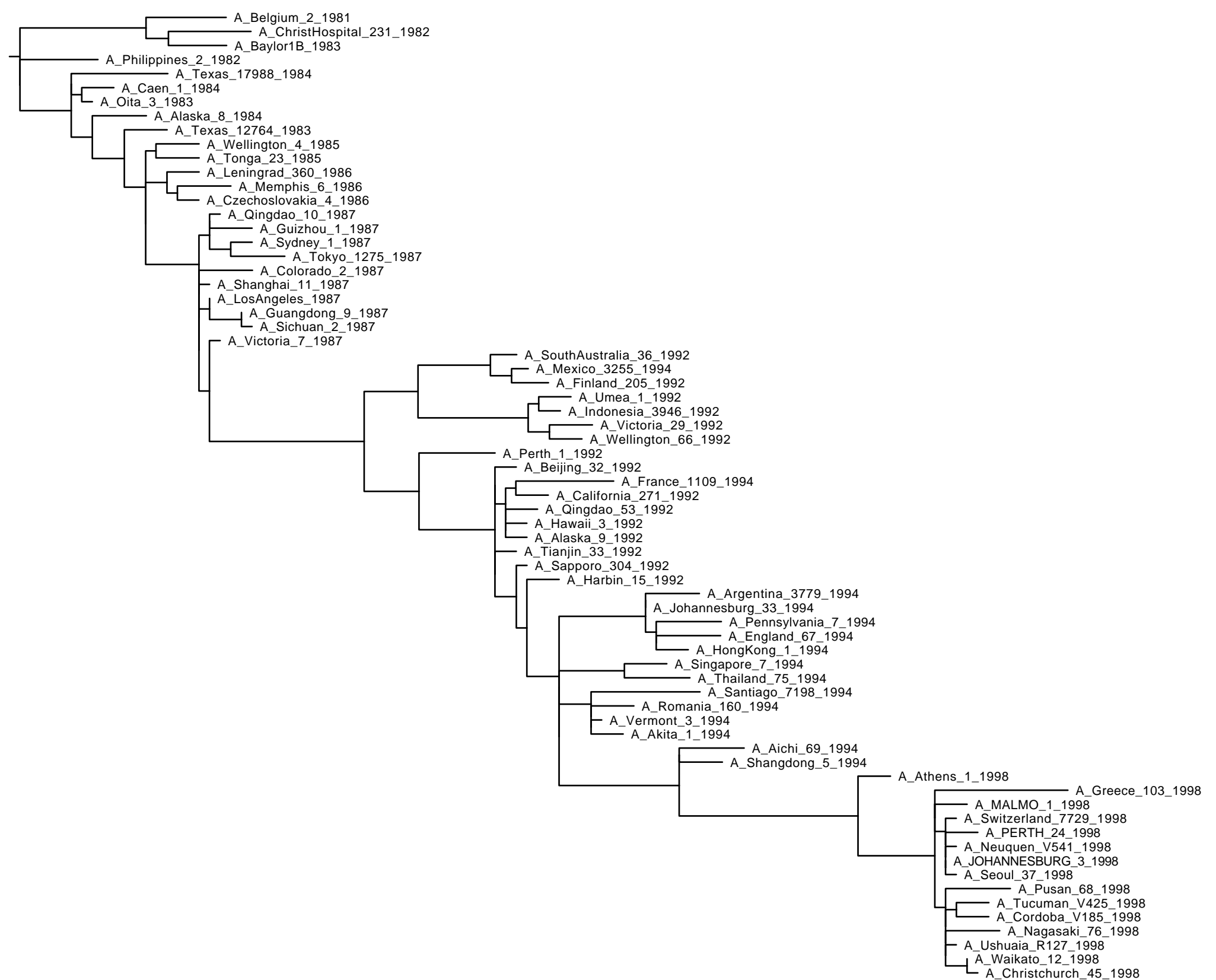

0.02

Supplement: Additional file 1: — Maximum likelihood phylogeny of the influenza A virus data. Maximum likelihood tree without the assumption of a molecular clock. Branch lengths depict the expected number of substitution per site. [file s12862-014-0163-6-S1.pdf]

**(a) Strict clock**

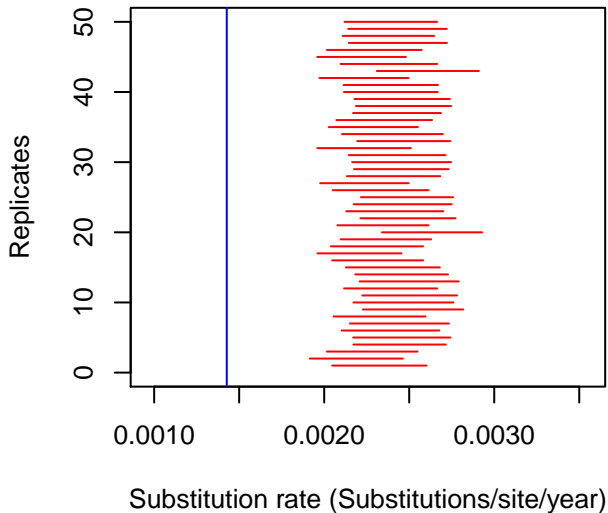

**(b) Strict clock**

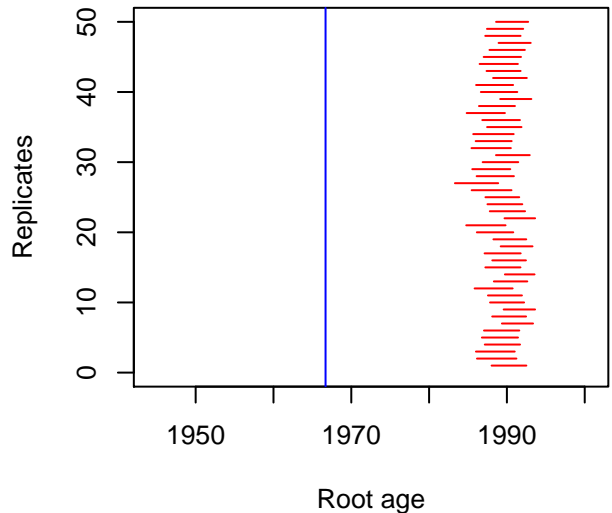

Supplement: Additional file 3: — 95% Bayesian confidence intervals of substitution rate and root age using the birth-death model and a strict clock in 50 simulated data sets. Confidence intervals of the nucleotide substitution rate (a) and root age (a) were inferred using BEAST for 50 replicates using a birth-death model prior on the phylogeny. Intervals that do not include the true value (blue line) are shown in red. [file s12862-014-0163-6-S3.pdf]

**(a) Linear regression**

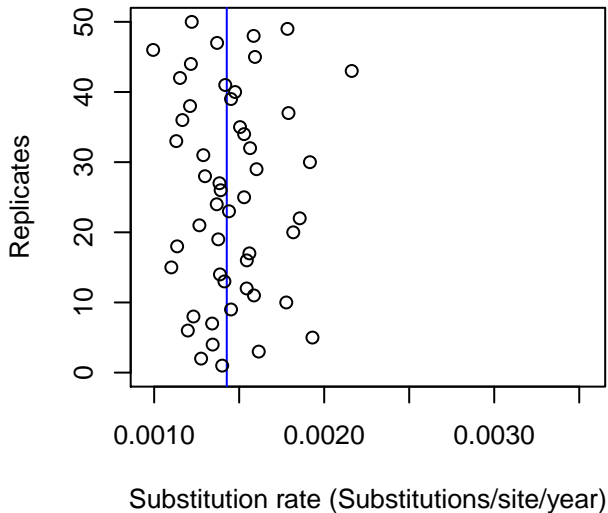

**(b) Linear regression**

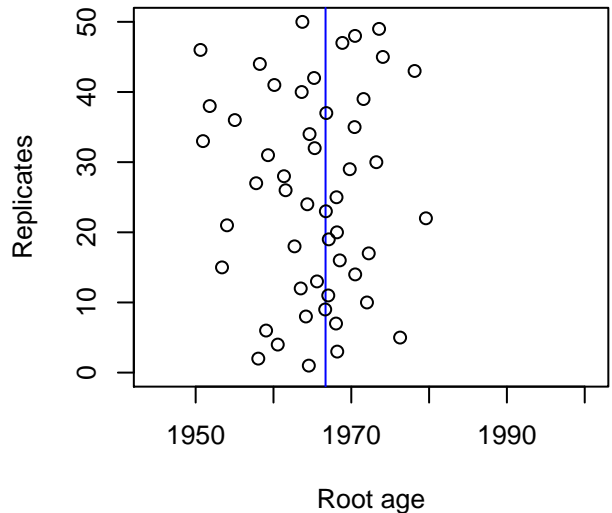

Supplement: Additional file 4: — Linear regression estimates of substitution rate and root age in 50 simulated data sets. Point estimates of the nucleotide substitution rate (a) and root age (b) using a root to tip linear regression of the expected number of substitutions per site and sampling dates. [file s12862-014-0163-6-S4.pdf]
