# Supplementary material for: Novel non-parametric models to estimate evolutionary rates and divergence times from heterochronous sequence data
Source: BMC Evol Biol. 2014 Jul 24;14:163. doi: 10.1186/s12862-014-0163-6 (PMC4222489; doi:10.1186/s12862-014-0163-6)
Supplement: Additional file 2: — Maximum likelihood phylogeny of the influenza B virus data. Maximum likelihood tree without the assumption of a molecular clock. Branch lengths depict the expected number of substitution per site. [file s12862-014-0163-6-S2.pdf]

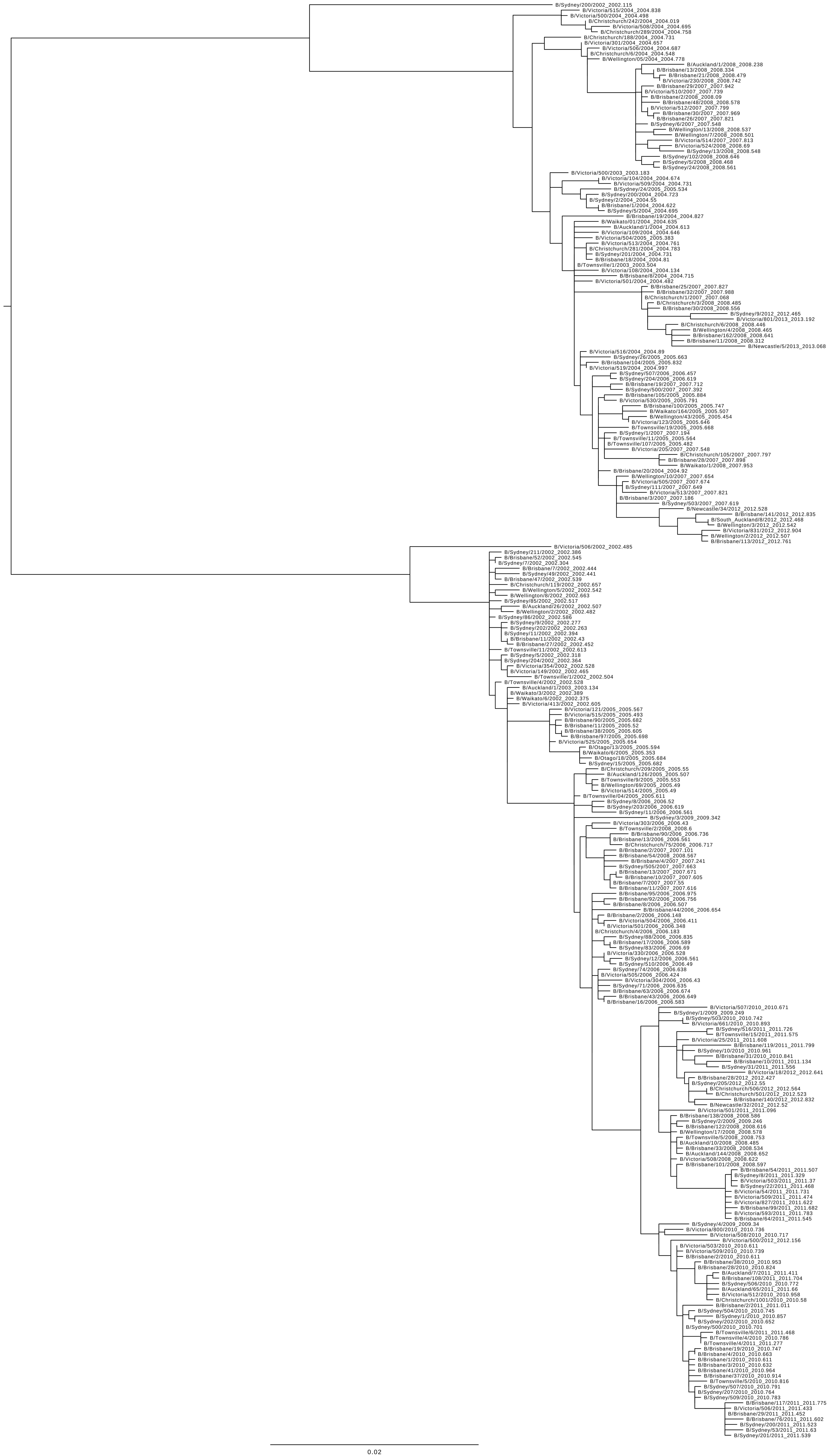
B/Sydney/200/2002_2002.115
B/Victoria/515/2004_2004.838
B/Victoria/500/2004_2004.498
B/Christchurch/242/2004_2004.019
B/Victoria/508/2004_2004.695
B/Christchurch/289/2004_2004.758
B/Christchurch/188/2004_2004.731
B/Victoria/301/2004_2004.657
B/Victoria/506/2004_2004.687
B/Christchurch/6/2004_2004.548
B/Wellington/05/2004_2004.778
B/Auckland/1/2008_2008.238
B/Brisbane/13/2008_2008.334
B/Brisbane/21/2008_2008.479
B/Victoria/230/2008_2008.742
B/Brisbane/29/2007_2007.942
B/Victoria/510/2007_2007.739
B/Brisbane/2/2008_2008.09
B/Brisbane/48/2008_2008.578
B/Victoria/512/2007_2007.799
B/Brisbane/30/2007_2007.969
B/Brisbane/26/2007_2007.821
B/Sydney/6/2007_2007.548
B/Wellington/13/2008_2008.537
B/Wellington/7/2008_2008.501
B/Victoria/514/2007_2007.813
B/Victoria/524/2008_2008.69
B/Sydney/13/2008_2008.548
B/Sydney/102/2008_2008.646
B/Sydney/5/2008_2008.468
B/Sydney/24/2008_2008.561
B/Victoria/500/2003_2003.183
B/Victoria/104/2004_2004.674
B/Victoria/509/2004_2004.731
B/Sydney/24/2005_2005.534
B/Sydney/200/2004_2004.723
B/Sydney/2/2004_2004.55
B/Brisbane/1/2004_2004.622
B/Sydney/5/2004_2004.695
B/Brisbane/19/2004_2004.827
B/Waikato/01/2004_2004.635
B/Auckland/1/2004_2004.613
B/Victoria/109/2004_2004.646
B/Victoria/504/2005_2005.383
B/Victoria/513/2004_2004.761
B/Christchurch/281/2004_2004.783
B/Sydney/201/2004_2004.731
B/Brisbane/18/2004_2004.81
B/Townsville/1/2003_2003.504
B/Victoria/108/2004_2004.134
B/Brisbane/8/2004_2004.715
B/Victoria/501/2004_2004.482
B/Brisbane/25/2007_2007.827
B/Brisbane/32/2007_2007.988
B/Christchurch/1/2007_2007.068
B/Christchurch/3/2008_2008.485
B/Brisbane/30/2008_2008.556
B/Sydney/9/2012_2012.465
B/Victoria/801/2013_2013.192
B/Christchurch/6/2008_2008.446
B/Wellington/4/2008_2008.465
B/Brisbane/162/2008_2008.641
B/Brisbane/11/2008_2008.312
B/Newcastle/5/2013_2013.068
B/Victoria/516/2004_2004.89
B/Sydney/26/2005_2005.663
B/Brisbane/104/2005_2005.832
B/Victoria/519/2004_2004.997
B/Sydney/507/2006_2006.457
B/Sydney/204/2006_2006.619
B/Brisbane/19/2007_2007.712
B/Sydney/500/2007_2007.392
B/Brisbane/105/2005_2005.884
B/Victoria/530/2005_2005.791
B/Brisbane/100/2005_2005.747
B/Waikato/164/2005_2005.507
B/Wellington/43/2005_2005.454
B/Victoria/123/2005_2005.646
B/Townsville/19/2005_2005.668
B/Sydney/1/2007_2007.194
B/Townsville/11/2005_2005.564
B/Townsville/107/2005_2005.482
B/Victoria/205/2007_2007.548
B/Christchurch/105/2007_2007.797
B/Brisbane/28/2007_2007.898
B/Waikato/1/2008_2007.953
B/Brisbane/20/2004_2004.92
B/Wellington/10/2007_2007.654
B/Victoria/505/2007_2007.674
B/Sydney/111/2007_2007.649
B/Victoria/513/2007_2007.821
B/Brisbane/3/2007_2007.186
B/Sydney/503/2007_2007.619
B/Newcastle/34/2012_2012.528
B/Brisbane/141/2012_2012.835
B/South_Auckland/8/2012_2012.468
B/Wellington/3/2012_2012.542
B/Victoria/831/2012_2012.904
B/Wellington/2/2012_2012.507
B/Brisbane/113/2012_2012.761
B/Victoria/506/2002_2002.485
B/Sydney/211/2002_2002.386
B/Brisbane/52/2002_2002.545
B/Sydney/7/2002_2002.304
B/Brisbane/7/2002_2002.444
B/Sydney/49/2002_2002.441
B/Brisbane/47/2002_2002.539
B/Christchurch/119/2002_2002.657
B/Wellington/5/2002_2002.542
B/Wellington/8/2002_2002.663
B/Sydney/85/2002_2002.517
B/Auckland/26/2002_2002.507
B/Wellington/2/2002_2002.482
B/Sydney/86/2002_2002.586
B/Sydney/9/2002_2002.277
B/Sydney/202/2002_2002.263
B/Sydney/11/2002_2002.394
B/Brisbane/11/2002_2002.43
B/Brisbane/27/2002_2002.452
B/Townsville/11/2002_2002.613
B/Sydney/5/2002_2002.318
B/Sydney/204/2002_2002.364
B/Victoria/354/2002_2002.528
B/Victoria/149/2002_2002.465
B/Townsville/1/2002_2002.504
B/Townsville/4/2002_2002.528
B/Auckland/1/2003_2003.134
B/Waikato/3/2002_2002.389
B/Waikato/6/2002_2002.375
B/Victoria/413/2002_2002.605
B/Victoria/121/2005_2005.567
B/Victoria/515/2005_2005.493
B/Brisbane/90/2005_2005.682
B/Brisbane/11/2005_2005.52
B/Brisbane/38/2005_2005.605
B/Brisbane/97/2005_2005.698
B/Victoria/525/2005_2005.654
B/Otago/13/2005_2005.594
B/Waikato/6/2005_2005.353
B/Otago/18/2005_2005.684
B/Sydney/15/2005_2005.682
B/Christchurch/209/2005_2005.55
B/Auckland/126/2005_2005.507
B/Townsville/9/2005_2005.553
B/Wellington/69/2005_2005.49
B/Victoria/514/2005_2005.49
B/Townsville/04/2005_2005.611
B/Sydney/8/2006_2006.52
B/Sydney/203/2006_2006.619
B/Sydney/11/2006_2006.561
B/Sydney/3/2009_2009.342
B/Victoria/303/2006_2006.43
B/Townsville/2/2008_2008.6
B/Brisbane/90/2006_2006.736
B/Brisbane/13/2006_2006.561
B/Christchurch/75/2006_2006.717
B/Brisbane/2/2007_2007.101
B/Brisbane/54/2008_2008.567
B/Brisbane/4/2007_2007.241
B/Sydney/505/2007_2007.663
B/Brisbane/13/2007_2007.671
B/Brisbane/10/2007_2007.605
B/Brisbane/7/2007_2007.55
B/Brisbane/11/2007_2007.616
B/Brisbane/95/2006_2006.975
B/Brisbane/92/2006_2006.756
B/Brisbane/8/2006_2006.507
B/Brisbane/44/2006_2006.654
B/Brisbane/2/2006_2006.148
B/Victoria/504/2006_2006.411
B/Victoria/501/2006_2006.348
B/Christchurch/4/2006_2006.183
B/Sydney/88/2006_2006.835
B/Brisbane/17/2006_2006.589
B/Sydney/83/2006_2006.69
B/Victoria/330/2006_2006.528
B/Sydney/12/2006_2006.561
B/Sydney/510/2006_2006.49
B/Sydney/74/2006_2006.638
B/Victoria/505/2006_2006.424
B/Victoria/304/2006_2006.43
B/Sydney/71/2006_2006.635
B/Brisbane/63/2006_2006.674
B/Brisbane/43/2006_2006.649
B/Brisbane/16/2006_2006.583
B/Victoria/507/2010_2010.671
B/Sydney/1/2009_2009.249
B/Sydney/503/2010_2010.742
B/Victoria/661/2010_2010.893
B/Sydney/516/2011_2011.726
B/Townsville/15/2011_2011.575
B/Victoria/25/2011_2011.608
B/Brisbane/119/2011_2011.799
B/Sydney/10/2010_2010.961
B/Brisbane/31/2010_2010.841
B/Brisbane/10/2011_2011.134
B/Sydney/31/2011_2011.556
B/Victoria/18/2012_2012.641
B/Brisbane/28/2012_2012.427
B/Sydney/205/2012_2012.55
B/Christchurch/506/2012_2012.564
B/Christchurch/501/2012_2012.523
B/Brisbane/140/2012_2012.832
B/Newcastle/32/2012_2012.52
B/Victoria/501/2011_2011.096
B/Brisbane/138/2008_2008.586
B/Sydney/2/2009_2009.246
B/Brisbane/122/2008_2008.616
B/Wellington/17/2008_2008.578
B/Townsville/5/2008_2008.753
B/Auckland/10/2008_2008.485
B/Brisbane/33/2008_2008.534
B/Auckland/144/2008_2008.652
B/Victoria/508/2008_2008.622
B/Brisbane/101/2008_2008.597
B/Brisbane/54/2011_2011.507
B/Sydney/8/2011_2011.329
B/Victoria/503/2011_2011.37
B/Sydney/22/2011_2011.468
B/Victoria/54/2011_2011.731
B/Victoria/509/2011_2011.474
B/Victoria/827/2011_2011.622
B/Brisbane/99/2011_2011.682
B/Victoria/593/2011_2011.783
B/Brisbane/64/2011_2011.545
B/Sydney/4/2009_2009.34
B/Victoria/800/2010_2010.736
B/Victoria/508/2010_2010.717
B/Victoria/500/2012_2012.156
B/Victoria/503/2010_2010.611
B/Victoria/509/2010_2010.739
B/Brisbane/2/2010_2010.611
B/Brisbane/38/2010_2010.953
B/Brisbane/28/2010_2010.824
B/Auckland/7/2011_2011.411
B/Brisbane/108/2011_2011.704
B/Sydney/506/2010_2010.772
B/Auckland/65/2011_2011.66
B/Victoria/512/2010_2010.958
B/Christchurch/1001/2010_2010.58
B/Brisbane/2/2011_2011.011
B/Sydney/504/2010_2010.745
B/Sydney/1/2010_2010.857
B/Sydney/202/2010_2010.652
B/Sydney/500/2010_2010.701
B/Townsville/6/2011_2011.468
B/Townsville/4/2010_2010.786
B/Townsville/4/2011_2011.277
B/Brisbane/19/2010_2010.747
B/Brisbane/4/2010_2010.663
B/Brisbane/1/2010_2010.611
B/Brisbane/3/2010_2010.632
B/Brisbane/41/2010_2010.964
B/Brisbane/37/2010_2010.914
B/Townsville/5/2010_2010.816
B/Sydney/507/2010_2010.791
B/Sydney/207/2010_2010.764
B/Sydney/509/2010_2010.783
B/Brisbane/117/2011_2011.775
B/Victoria/506/2011_2011.433
B/Brisbane/29/2011_2011.452
B/Brisbane/76/2011_2011.602
B/Sydney/200/2011_2011.523
B/Sydney/53/2011_2011.63
B/Sydney/201/2011_2011.539
0.02
